# Aerosol delivery of dry powder synthetic lung surfactant to surfactant-deficient rabbits and preterm lambs on non-invasive respiratory support

**DOI:** 10.12688/gatesopenres.12899.2

**Published:** 2019-03-14

**Authors:** Frans J. Walther, Monik Gupta, Michael M. Lipp, Holly Chan, John Krzewick, Larry M. Gordon, Alan J. Waring

**Affiliations:** 1 Department of Pediatrics, Los Angeles Biomedical Research Institute at Harbor-UCLA Medical Center, Torrance, California, 90502, USA; 2 Acorda Therapeutics Inc., Chelsea, Massachusetts, 02150, USA; 3 Department of Medicine, Los Angeles Biomedical Research Institute at Harbor-UCLA Medical Center, Torrance, California, 90502, USA

**Keywords:** Dry Powder synthetic lung surfactant, Surfactant Protein B, Super Mini-B, Respiratory Distress Syndrome, Fourier-Transform InfraRed spectrometry, Captive Bubble Surfactometry, lung lavage rabbit model, preterm lambs

## Abstract

**Background**: The development of synthetic lung surfactant for preterm infants has focused on peptide analogues of native surfactant proteins B and C (SP-B and SP-C). Non-invasive respiratory support with nasal continuous positive airway pressure (nCPAP) may benefit from synthetic surfactant for aerosol delivery.

**Methods**: A total of three dry powder (DP) surfactants, consisting of phospholipids and the SP-B analogue Super Mini-B (SMB), and one negative control DP surfactant without SMB, were produced with the Acorda Therapeutics ARCUS® Pulmonary Dry Powder Technology. Structure of the DP surfactants was compared with FTIR spectroscopy, *in vitro* surface activity with captive bubble surfactometry, and *in vivo* activity in surfactant-deficient adult rabbits and preterm lambs. In the animal experiments, intratracheal (IT) aerosol delivery was compared with surfactant aerosolization during nCPAP support. Surfactant dosage was 100 mg/kg of lipids and aerosolization was performed using a low flow inhaler.

**Results: **FTIR spectra of the three DP surfactants each showed secondary structures compatible with peptide folding as an α-helix hairpin, similar to that previously noted for surface-active SMB in other lipids. The DP surfactants with SMB demonstrated *in vitro* surface activity <1 mN/m. Oxygenation and lung function increased quickly after IT aerosolization of DP surfactant in both surfactant-deficient rabbits and preterm lambs, similar to improvements seen with clinical surfactant. The response to nCPAP aerosol delivery of DP surfactant was about 50% of IT aerosol delivery, but could be boosted with a second dose in the preterm lambs.

**Conclusions:** Aerosol delivery of DP synthetic surfactant during non-invasive respiratory support with nCPAP significantly improved oxygenation and lung function in surfactant-deficient animals and this response could be enhanced by giving a second dose. Aerosol delivery of DP synthetic lung surfactant has potential for clinical applications.

## Introduction

Lung surfactant is a lipid-protein mixture that is synthesized by alveolar type II cells and secreted into the alveoli to reduce surface tension at the air-liquid interface. Lack or dysfunction of lung surfactant leads to respiratory failure, as seen in very preterm infants with respiratory distress syndrome (RDS) due to lung immaturity and surfactant deficiency and in (near-) term infants with surfactant dysfunction due to meconium aspiration syndrome. Mammalian lung surfactant harvested by lavage or lung extraction has been instrumental in reducing mortality and morbidity in preterm infants with RDS ^1^. However, animal surfactant has some major drawbacks, such as limited supplies, complex production and sterilization processes, high production costs, and the need for intratracheal (IT) delivery. This has stimulated the design and testing of synthetic lung surfactant formulations with peptide mimics of the pivotal surfactant protein B (SP-B) and the less essential surfactant protein C (SP-C) ^2^. The realization that the etiology of chronic lung injury (a.k.a. bronchopulmonary dysplasia, BPD) is multifactorial and intubation and mechanical ventilation are associated with a higher risk of BPD has led to a preference for non-invasive respiratory support with nasal continuous positive airway pressure (nCPAP) in preterm infants ^3^. Therefore, clinical neonatal care might benefit from a synthetic lung surfactant formulation that can be delivered by inhalation and without the need for endotracheal intubation of a preterm infant.

**Figure 1.**
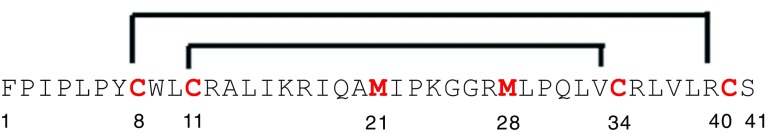
Sequence for Super Mini-B (SMB). SMB (41 amino-acid residues; 1-letter amino-acid notation) with the N- and C-terminal Phe-1 and Ser-41 are indicated, and also the sulfur-containing cysteines (Cys-8, Cys-11, Cys-34, Cys-40) and methionines (Met-21 and Met-28) in red. The two disulfide-linkages are shown between Cys-11 and Cys-34 and between Cys-8 and Cys-40.

Using 3D-saposin motifs for SP-B predicted from homology modeling, we produced truncated SP-B peptide mimics with an α-helix hairpin structure and high surface activity in surfactant-deficient animals ^2, 4^. One of these ‘short-cut’ peptides is Super Mini-B (SMB), a 41-residue SP-B mimic ( Figure 1) based on the amino-acid sequence, structure, folding and association of native SP-B (79-residues). Surface activity of SMB is similar to that of its parent protein ^4– 6^. SMB consists of the N-terminal (~residues 1-25) and C-terminal α-helices (~residues 63–78) of native SP-B ( Figure 1) and is connected with a –PKGG– turn to form a α-helix hairpin (α-helix/turn/α1-helix, a.k.a. αtα) ^7^. SMB has two disulfide bonds that lie close together (i.e., Cys-8 to Cys-77 and Cys-11 to Cys-71) and covalently link both α-helices to reinforce the formation of an α-helix hairpin. The structure of SMB has been confirmed with spectroscopy, homology modeling and molecular dynamics simulations in lipid mimics and lipid bilayers ^5, 6, 8^. Formulated with surfactant lipids, SMB has demonstrated stability of the α-helix hairpin and continuous high surface activity in fresh and stored preparations ^4, 5, 8^. In this context, it should be noted that previous work demonstrated that aerosol delivery of synthetic lung surfactant with SP-B and SP-C peptides could improve oxygenation and lung function in spontaneously breathing, lavaged, surfactant-deficient rabbits supported with nCPAP ^9^. Nevertheless, the above highly active surfactant formulations were all liquids, may require cold-chain technology to ensure shelf-life and may therefore not be practical in low-resource settings.

In the present study, we report the development of surface-active dry powder (DP) synthetic lung surfactant formulations, their *in vitro* surface activity in the captive bubble surfactometer and their *in vivo* surface activity following aerosol delivery in spontaneously breathing, surfactant-deficient rabbits and preterm lambs supported with nCPAP. These DP surfactants consisted of 2-3 phospholipids combined with a SP-B peptide mimic (SMB) and necessary additives, and one DP surfactant formulation without SMB as negative control. Although SMB has primarily been studied in liquids (see above), our finding that SMB maintains its engineered α-helix turn in a wide range of environments ^5, 8^ suggests that this SP-B mimic may also retain its structure and surface activity in the anhydrous state characteristic of dry powder aerosols.

## Methods

### Preparation of DP synthetic lung surfactant formulations for aerosol delivery

DP synthetic surfactant powders were designed and formulated at Acorda Therapeutics, utilizing its ARCUS ^®^ Pulmonary Dry Powder Technology. The challenge that was overcome via the utilization of the ARCUS ^®^ technology was to produce phospholipid-based DP lung surfactant formulations, with and without SP-B peptide mimetic, that both replicated the fluidizing surface activity of endogenous lung surfactant yet were both physically and chemically stable as well as dispersible and aerosolizable in a solid-state DP form, in contrast to the liquid-based forms currently utilized for surfactant replacement therapy. An extensive screening process ^10^ was conducted utilizing various combinations of phospholipids, fatty acids and stabilizing excipients that resulted in a lead set of optimized peptide-free candidate DP synthetic surfactant powders. SP-B peptide mimic-containing formulations were later produced utilizing this lead set of powders. Lead DP synthetic surfactant powders were formulated by adding the following components to the organic solvent used for spray drying: 49 wt% DPPC and 21 wt% POPG-Na (Avanti Polar Lipids, Alabaster, AL); an optional third phospholipid, e.g., 7 wt% POPC (Avanti Polar Lipids, Alabaster, AL 35007); an excipient, e.g. polyglycitol Stabilite™ SD-30 (Grain Processing Corp., Muscatine, IA 52761); an optional fatty acid, e.g., 5 wt% palmitic acid (PA) (Sigma-Aldrich Co, Saint Louis, MO 63103); 2 wt% NaCl (Sigma-Aldrich Corporation, Saint Louis, MO 63103); and optionally 3 wt% of a SP-B peptide mimic (SMB); with all components amounting to 100 wt%.

SMB was synthesized at the Los Angeles Biomedical Research Institute using a standard Fmoc protocol with a Symphony Multiple Peptide Synthesizer (Protein Technologies, Inc., Tucson, AZ 87514) or a CEM Liberty microwave synthesizer (CEM Corporation, Mathews, NC 28104), cleaved-deprotected, purified using reverse phase HPLC, freeze-dried, and had its mass confirmed by MALDI TOF mass spectrometry and concentration quantitated using UV absorbance ^4, 5^.

Respirable dry synthetic surfactant particles were produced at Acorda Therapeutics using a GEA Niro PSD-1 spray dryer (Niro Inc., Copenhagen, Denmark) or Buchi B-290 mini (Buchi Corpration, New Castle, DE 19720) spray dryer. After spray drying, the synthetic surfactant powders were filled into size 00 capsules. Testing of aerosol properties and solid-state properties of the DP surfactant formulations included particle sizing, X-ray diffraction, thermogravimetric analysis and differential scanning calorimetry. Respirable dry particles with a mass median aerodynamic diameter of between 1 and 4 microns were preferred. A set of lead optimized DP synthetic surfactant formulations were then provided to Los Angeles Biomedical Research Institute in a blinded manner for further *in vitro* and *in vivo* characterization.

Next, three DP synthetic surfactants containing SMB were chosen for more extensive *in vitro* and *in vivo* testing and compared to a lipid control powder without SMB (DPPC:POPG-Na:SD-30:NaCl 49:21:28:2 wt:wt) as a negative control (DP-C) and the clinical surfactant Curosurf ^^®^^, a porcine surfactant containing both SP-B and SP-C, as a positive control, where appropriate. The three surfactant formulations with SMB were: DPPC:POPG-Na:SD-30:SP-B:NaCl 49:21:25:3:2 wt:wt (DP-2L); DPPC:POPG-Na:POPC:SD-30:SP-B:NaCl 49:21:7:18:3:2 wt:wt (DP-3L); and DPPC:POPG-Na:SD-30:PA:SP-B:NaCl 49:21:20:5:3:2 (DP-PA).

### Aerosol delivery

Because there is currently no commercially available system designed specifically for inhalation therapy during nCPAP, a proprietary DP delivery device was designed and produced by Acorda Therapeutics (Chelsea, MA 02150). This low flow aerosolization chamber (LFAC) consists of a cylindrical chamber with one or more holes at one end that can accommodate a perforated capsule containing powder and disperse it at low flow rates (4–10 l/min) and can also be incorporated into a ventilatory system like nCPAP in lieu of being utilized as a stand-alone device. Depending on the flow rate, DP surfactant is delivered as a fine mist over 1–3 minutes per capsule filled with about 30 mg of surfactant. Dosing was based on the lipid content of the DP surfactant and amounted 100 mg/kg bodyweight. In consideration of the potential applications of this work, in addition to its efficacy in aerosolizing and delivering DP synthetic surfactants, additional design criteria for the device included the following (1) simplicity of design and use, (2) minimum number of parts, and (3) low cost of goods/manufacture.

### Attenuated-Total-Reflectance Fourier-transform infrared (ATR-FTIR) spectrometry

ATR-FTIR spectra were recorded at 37°C using a Bruker Vector 22 FTIR spectrometer (Pike Technologies, Fitchburg, WI 53719) with a deuterium triglyceride sulfate (DTGS) detector. The spectra were averaged over 256 scans at a gain of 4 and a resolution of 2 cm ^-1^ ^5^. For FTIR spectra of SMB in DP synthetic lung surfactant, the powder was solvated with deuterated water (D _2_O) and transferred onto a germanium ATR crystal. The aqueous solvent was then removed by flowing nitrogen gas over the sample to produce a thick lipid-peptide (lipid:peptide ratios of 10:0.3, mole:mole) ^5^. The multilayer film was then hydrated to ≥35% with deuterated water vapor in nitrogen for 1 h before acquiring the spectra ^11^. The spectra for the SMB peptide in the lipid film were obtained by subtracting the spectrum of a peptide-free control sample from that of the peptide-bound sample. The relative amounts of α-helix, β-turn, β-sheet, or random (disordered) structures in lipid-peptide films were estimated using Fourier deconvolution (GRAMS AI 8, version 8.0, Thermo Fisher Scientific, Waltham, MA 02451). The respective areas of component peaks were calculated using curve-fitting software (Igor Pro, version 1.6, Wavemetrics, Lake Oswego, OR 97035) ^12^. FTIR frequency limits were: α-helix (1662-1650 cm ^-1^), β-sheet (1637-1613 cm ^-1^), turn/bend (1682-1662 cm ^-1^), and disordered or random (1650-1637 cm ^-1^) ^13^.

### Captive bubble surfactometry

Surface activity of the DP surfactant formulations was measured with a captive bubble surfactometer built by Schürch and coworkers ^14, 15^. DP surfactant formulations were dissolved in distilled water at a concentration of 35 mg/mL and quasi-static and dynamic compression cycling of the air bubble was performed at an average surfactant lipid concentration of 70 µg/mL in the bubble chamber (~1.0 mL volume) ^5, 16^. All measurements were performed in quadruplicate.

### *In vivo* experiments: surfactant-deficient rabbits

Animal experiments were performed according to protocols that were reviewed and approved by the Institutional Animal Care and Use Committee of the Los Angeles Biomedical Research Institute at Harbor-UCLA Medical Center (LA BioMed protocol # 020645) ^16^. All procedures and anesthesia were in accordance with the American Veterinary Medical Association (AMVA) guidelines. Treatment allocation was done by dynamic randomization and minimum sample size per treatment group was based on effect size ^16^.

Lung lavaged rabbits are surfactant-deficient for at least 6–8 hours, but need respiratory support and surfactant treatment for survival. A total of 30 young adult, male New Zealand white rabbits with a weight of 1.0–1.4 kg were purchased from IFPS Inc. (Norco, CA 92860). Veterinary care; anesthesia, sedation, and muscle paralysis; and provision of maintenance fluid have been reported previously ^5, 16^. After inducing anesthesia, a venous line was placed via a marginal ear vein, an orotracheal tube was inserted, mechanical ventilation was started, and a carotid arterial line was inserted via a small incision in the skin of the anterior neck. Heart rate, arterial blood pressures and rectal temperature were monitored continuously (Labchart ^®^ Pro, ADInstruments Inc., Colorado Springs, CO 80906). Respiratory support was provided with volume-controlled ventilation (Harvard Apparatus, Holliston, MA 01746) using a tidal volume 7.5 mL/kg, a positive end-expiratory pressure of 3 cm H _2_O, an inspiratory/expiratory ratio of 1:2, 100% oxygen, and a respiratory rate sufficient to maintain the partial pressure of carbon dioxide (PaCO _2_) at ∼40 mmHg ^5, 16^. Airway flow and pressures and tidal volume were monitored with a pneumotachograph (Hans Rudolph Inc., Shawnee, KS 66227) connected to the orotracheal tube during mechanical ventilation. After a partial pressure of oxygen in arterial blood (PaO _2_) >500 mmHg was reached at a peak inspiratory pressure <15 cm H _2_O in 100% oxygen, surfactant-deficiency was obtained with repeated lung lavages with 30 mL/kg of warmed normal saline. When the PaO _2_ was stable at <100 mmHg (average four lavages), one of two surfactant treatment approaches was chosen in a random fashion: (1) insufflation of surfactant via the orotracheal tube using the LFAC device with continuation of mechanical ventilation (n=13), or (2) extubation after establishment of adequate spontaneous breathing, followed by placement on nasal continuous positive airway pressure (nCPAP) using a F&P bubble CPAP system (Fisher & Paykel Healthcare Inc., Irvine, CA 92618) with short home-made nasal prongs and insufflation of surfactant with the LFAC device at its lowest possible flow setting via the nCPAP system ^9^ (n=17). DP surfactant was administered at a dose of 100 mg lipids/kg body weight. Oxygenation was followed by measuring arterial pH and blood gases and, if the rabbit was intubated and mechanically ventilated, dynamic lung compliance at 15 min intervals over a 2 h period. At 2 h after surfactant administration, rabbits were euthanized with 100 mg/kg of pentobarbital intravenously. Animals supported on nCPAP were re-intubated via a tracheotomy and mechanically ventilated to measure dynamic lung compliance. Oxygenation and dynamic lung compliance were used as end-points.

### *In vivo* experiments: preterm lambs

Preterm lambs at 135 days of gestation (term is 150 d) are surfactant-deficient, but able to breathe spontaneously while supported with CPAP delivered with nasal prongs (nCPAP) ^17, 18^. A total of 12 date-mated pregnant ewes with twin pregnancies were treated with 12 mg of betamethasone by intramuscular injection 48 and 24 h prior to delivery by cesarean section. Anesthesia of the ewes was induced with 10 mg/kg of ketamine IM and maintained with oxygen and isoflurane after placement of a cuffed endotracheal tube and starting mechanical ventilation. The uterus was then exposed through a ventral midline incision, the fetus(es) were located and their legs and abdomen were exposed through a hysterotomy, followed by insertion of an arterial and a venous umbilical catheter, exteriorization, insertion of an orotracheal tube (ID 4.0 mm) for mechanical ventilation, and cutting of the umbilical cord. Prior to the cesarean section, heparinized blood was collected from the jugular vein of the ewe to be administered to the lambs in case of hypotension or blood loss. After the cesarean section, ewes were euthanized by infusing 100 mg/kg of pentobarbital IV.

The lambs were dried, weighed, placed in a warmer on a heating pad and under heating lamps, and started on mechanical ventilation after a sustained inflation at 40 cm H _2_O pressure for 20 sec. Initial ventilator settings were: peak inspiratory pressure (PIP) of 30 cm H _2_O, positive end-expiratory pressure (PEEP) of 8 cm H _2_O, inspiratory time of 0.4 sec, and respiratory rate of 60/min. PIP and respiratory rate were adjusted to achieve tidal volumes of 6 (5–10) mL/kg, a pH >7.20 and PaCO _2_ <60 mmHg. Both nostrils of the lambs were sprayed with 10% lidocaine spray and custom-made binasal prongs (shortened Portex tracheal tubes, ID 3.0 mm) were inserted 6–7 cm for CPAP delivery ^17^. Spontaneous breathing was stimulated with 20 mg/kg of caffeine and 5 mg/kg of doxapram IV, followed by continuous infusion of 2.5 mg/kg/h of doxapram. Heart rate, arterial blood pressure, oxygen saturation (pulse oximetry) and rectal temperature were recorded continuously. Anesthesia was provided by administering 30 mg/kg/h of propofol IV as needed. Maintenance fluid consisted of Lactated Ringer’s solution at a rate of 10 mL/kg/h. Airway flow and pressures and tidal volume were monitored during mechanical ventilation with a pneumotachograph (Hans Rudolph Inc., Shawnee, KS 66227).

Respiratory failure was monitored by observing oxygen saturation and confirmed by PaO _2_ levels <150 mmHg in 100% oxygen, at which time lambs were treated with synthetic DP lung surfactant. Surfactant was given either (1) with the LFAC device attached to the orotracheal tube, followed by extubation to nasal intermittent positive pressure ventilation (NIPPV) and then to nCPAP (heated and humidified bubble CPAP with PEEP 8-9 cm H _2_O and 100% oxygen), or (2) during nCPAP (after extubation and transition via NIPPV) with the LFAC device attached to one of the nasal prongs of the CPAP system and a short period of mononasal CPAP support, or (3) same as (2) with a second dose of surfactant 1 h after the initial dose. So, the location of aerosol delivery of synthetic DP lung surfactant (IT or via nCPAP) and the number of surfactant doses during nCPAP (1 or 2) were the only differences between the three treatment groups. Aerosol delivery of surfactant powder usually took about 15 min from start to finish. After surfactant treatment, arterial pH and blood gas measurements were done every 15–30 minutes. At 3 h after surfactant treatment, the lambs were sacrificed with 100 mg/kg of pentobarbital IV. After euthanasia, the thorax was opened by a midline incision, an endotracheal tube (ID 4.0 mm) was re-inserted, and a pressure volume curve was recorded. End-points of the experiment included: gas exchange (arterial pH and blood gases) and pulmonary mechanics (dynamic compliance during mechanical ventilation, postmortem pressure-volume curve).

### Statistical analysis

All data are expressed as mean ± SEM. Student's t-tests were used for comparisons of discrete data points and functional data were analyzed with one-way analysis of variance (ANOVA) with Scheffe’s post-hoc test. Differences with a P value <0.05 were considered statistically significant.

## Results

### DP synthetic lung surfactant formulations

Aerosol properties and solid-state properties of the DP surfactant formulations are summarized in Table 1. Geometric particle size (gPSD) and fine particle fraction (FPF) were well within the margins for respiratory particles. The FPF of DP-2L remained relatively constant across all stability time points whereas a drop in FPF was observed for DP-PA and DP-3L. The solid-state properties are similar among the various formulations. The DP formulations are semi-crystalline with a characteristic diffraction peak at 21° 2theta, which is attributed to the presence of phospholipids in a bilayer structure. Residual solvent (ethanol and water) content is less than 1.5 wt%. The DP formulations contain phase transitions at ~45–53°C, calculated as the intercept of a step transition, and 61–67°C, calculated as a peak extremum of an endotherm. The stability data of these formulations indicate that the powders would be viable for long-term storage.

**Table 1.** Aerosol properties and solid state properties of each of the DP surfactant formulations tested. The powders were spray dried, filled into size 00 capsules, and packaged in heat-sealable pouches. Accelerated stability testing was conducted at 40°C. The stability of these formulations indicate that the powders would be viable for long-term storage.

Description	Condition		gPSD μm	Size 00 FPF <5.6 μm (%)	Size 00 FPF <3.4 μm (%)			DSC	
						XRPD	TGA-120 (%)	Low T1 (°CC)	Low T2 (°CC)
**DP-2L**: DPPC:POPG-Na:SP-B: SD-30:NaCl (49:21:3:25:2)	t=0		6	74	66	SC-D	1.05	44.5	61.4
	40°C	2 wk		74	61	Not tested			
		3 mth		73	58				
		6 mth		67	52				
	5°C	12 mth		66	57				
**DP-PA**: DPPC:POPG-Na:PA:SP-B: SD-30:NaCl (49:21:5:3:20:2)	t=0		3.2	82	77	SC-D	1	48.5	66.5
	40°C	1 mth		75	62	Not tested			
		3 mth		55	39				
**DP-3L**: DPPC:POPG: POPC:SP-B: SD-30:NaCl (49:21:7:3:18:2)	t=0		4.1	73	65	SC-D	1.34	53.4	62.8
	40°C	1 mth		60	44	Not tested			
		3 mth		53	34				
**DP-C**: DPPC:POPG-Na:SD-30: NaCl (49:21:28:2)	t=0		4.5	67	58	SC-D	1.22	45.0	61.8

gPSD, average geometric particle size (gPSD) in microns (d50); FPF, fine particle fraction of the total dose < 5.6 and/or 3.4 microns; XRPD, X-ray powder diffraction; SC-D, semicrystalline material with diffraction peaks attributed to the presence of phospholipids in a bilayer structure; DSC,differential scanning calorimetry; Low T1, characteristic temperature(s) of the first thermal event(s) observed during a DSC scan at 20 °C/min; Low T2, characteristic temperature(s) of the second set of thermal event(s) observed during a DSC scan at 20°C/min; TGA-120 (%), volatiles loss by 120°C.

### FTIR results

**Figure 2.**
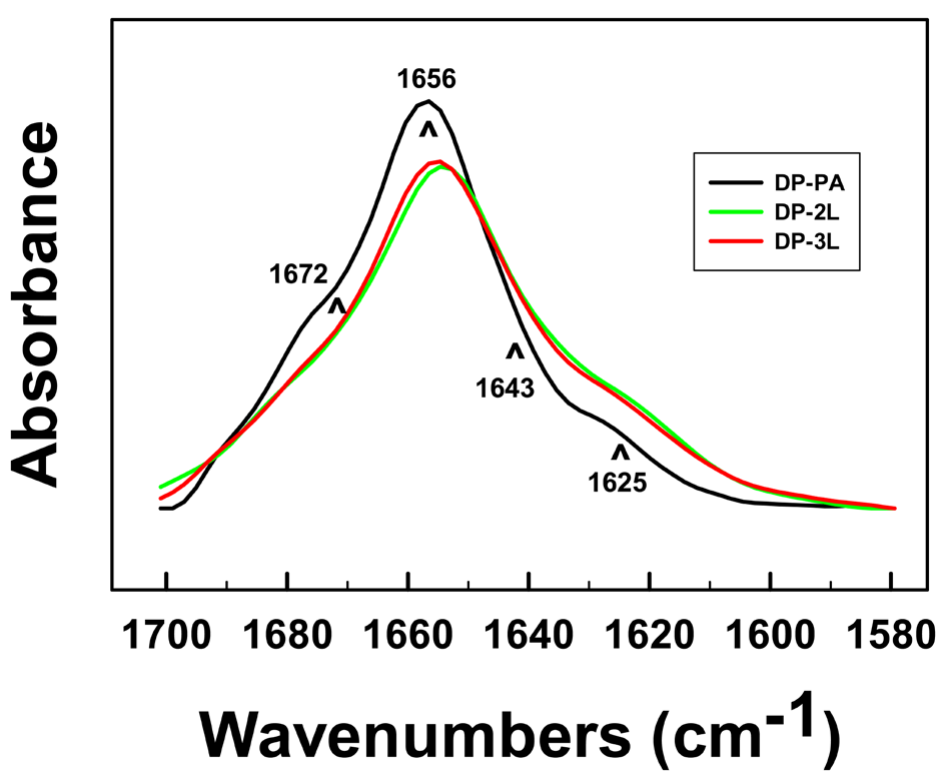
FTIR spectra of Super Mini-B (SMB) in dry powder (DP) synthetic surfactant lipid formulations, including DP-2L (green line), DP-3L (red line) and DP-PA (black line). Attenuated transform infrared (ATR-FTIR) spectra for each DP preparation are plotted using the same arbitrary absorbance units (See Methods and Results). Each IR spectrum shows a dominant α-helical component centered at the midpoint of the 1662-1650 cm ^-1^ band (e.g., see arrow in the DP-PA spectrum at 1656 cm ^-1^). Minor contributions are also indicated by arrows on the DP-PA spectrum at the 1625 cm ^-1^ band due to ß-sheet, at the 1672 cm ^-1^ band due to turns and bends, and at the 1643 cm ^-1^ band due to disordered or random conformations. The DP-3L spectrum is based on a representative sample of two productions. Peptide concentrations were 3.0 wt% for lipid-peptide ensembles. The areas under each absorbance curve are normalized using a SigmaPlot 14.0® macro.

The secondary structures for SMB in DP-2L, DP-3L, and DP-PA were studied with conventional ^12^C-FTIR spectroscopy of samples solvated in D _2_O. Representative FTIR spectra of the amide I band for SMB in these environments were all similar ( Figure 2), each showing a primary component centered at ~1654-1656 cm ^-1^, with a small low-field shoulder at ~1622-1626 cm ^-1^. Because prior FTIR studies of proteins and peptides ^13, 19^ have assigned bands in the range of ~1650-1659 cm ^-1^ as α-helical, while those at ~1613-1637 cm ^-1^ denote β-sheet, SMB probably assumes α-helical and β-sheet structures and possibly other conformations in these environments. Self-deconvolutions of the Figure 2 spectra confirmed that SMB is polymorphic, primarily adopting α-helix but with significant contributions from β-sheet, loop-turn and disordered components ( Table 2). Interestingly, the relative proportions of secondary conformations determined from FTIR spectra of SMB (i.e., α-helix > loop-turn ~ disordered ~ β-sheet) in both lipid mimetics and surfactant lipids of varying polarity are all comparable ( Table 2) ^5, 8^, suggesting overall stability of the SMB structure that is remarkably conserved. It should also be noted that the proportions of these secondary conformations are entirely compatible with SMB principally assuming an α-helix hairpin ^8^, and which has been shown to be surface active in *in vivo* experiments involving intratracheal instillation of liquid surfactants ^5^. 

**Table 2.** Spectroscopic proportions of the secondary structure ^a, b^ for SMB in DP surfactant lipids.

System	% Conformation ^a^			
	α-Helix	Loop-Turn	β-Sheet	Disordered
**DP-2L:** DPPC:POPG-Na:SD-30:SP-B:NaCl (49:21:25:3:2 wt:wt)	46.88	26.01	15.75	11.36
**DP-3L:** DPPC:POPG-Na:POPC:SD-30:SP-B:NaCl (49:21:7:18:3:2 wt:wt)	45.94	25.64	11.47	16.95
**DP-PA:** DPPC:POPG-Na:PA:SD-30:SP-B:NaCl (49:21:5:20:3:2 wt:wt)	48.12	26.56	14.86	10.46

^a^Tabulated results are means from four closely-reproduced separate determinations for each condition and spectral type. Powder was solvated with D _2_O and dried onto the germanium. FTIR sample crystal using a stream of dry nitrogen gas. The dried film was then hydrated with deuterated water vapor for one hour prior to spectral measurement. ^b^See Figure 2. ATR-FTIR spectra were estimated for proportions of the secondary structure for SMB in surfactant lipid films using self-deconvolution of the peptide amide I band (see Methods).

### Captive bubble surfactometry

**Figure 3.**
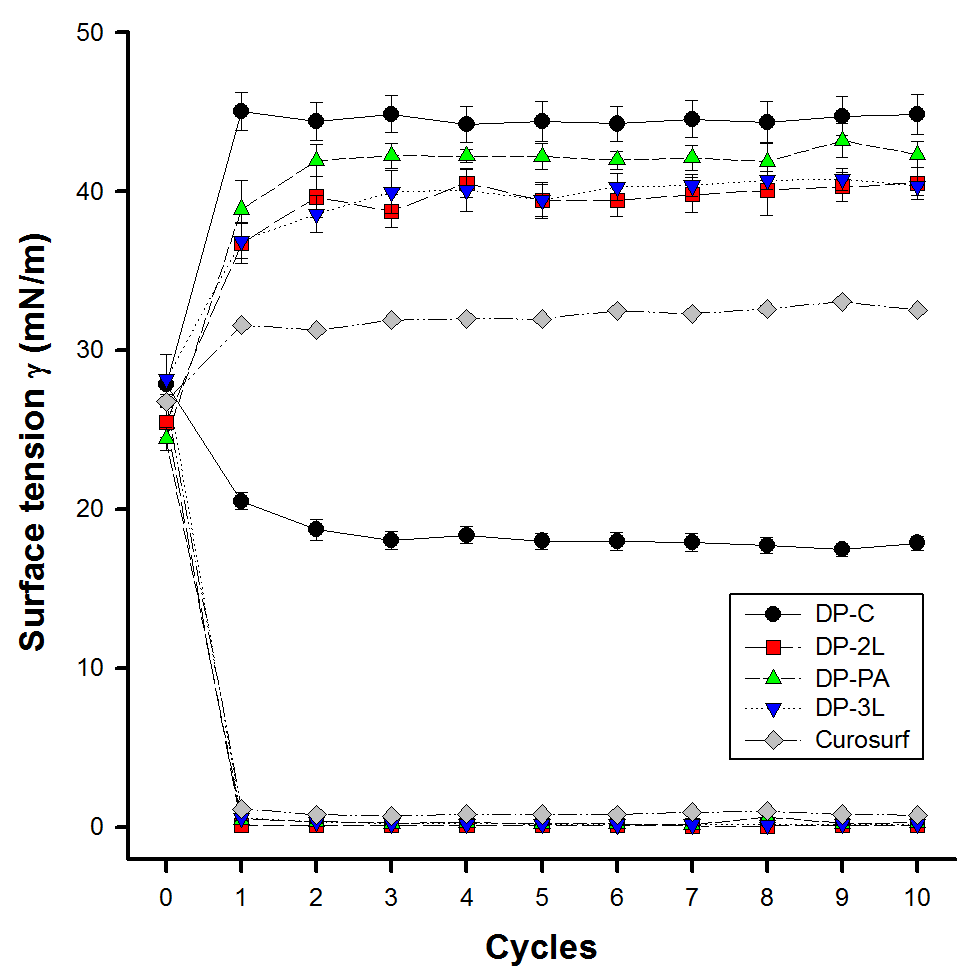
Quasi-static cycling in the captive bubble surfactometer. Surface activity of dry powder (DP) surfactant powders with 3 wt% of Super Mini-B (SMB) (DP-2L, DP-3L and DP-PA), the negative control with lipids only (DP-C), and the clinical surfactant Curosurf ^®^ as positive control. The lower part of each curve indicates minimum surface tension and the upper part indicates maximum surface tension during 10 quasi-static compression-expansion cycles. Minimum surface tension values of DP-2L, DP-3L and DP-PA surfactant were similar to those of Curosurf ^®^. Values are mean ± SEM of N ≥ 4. Error bars for the Curosurf ^®^ data were small and contained within the data point markers.

All three DP surfactant formulations with 3 wt% SMB, i.e. DP-2L, DP-3L and DP-PA, exhibited a minimum surface tension below 1 mN/m during quasi-static cycling and were comparable with the positive control, i.e. Curosurf ^®^, indicating that there was no loss in *in vitro* surface activity during the surfactant production process ( Figure 3). Minimum surface tension values of DP surfactant without SMB (DP-C), our negative control, far exceeded those of the surfactant formulations with SMB and amounted to ~18 mN/m (p<0.001), comparable to equivalent non-spray dried surfactant lipid formulations.

**Figure 4.**
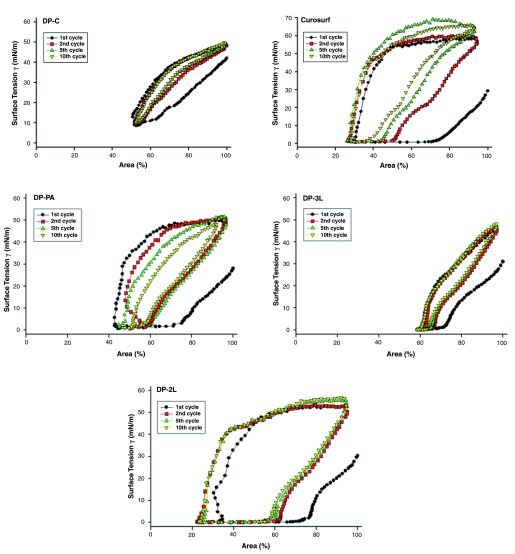
Dynamic cycling in the captive bubble surfactometer. Dynamic (20 cycles/min with approximately 20% overcompression) surface tension-area relationships of DP surfactant powders with 3 wt% of SMB (DP-2L, DP-3L and DP-PA), the negative control with lipids only (DP-C), and the clinical surfactant Curosurf ^®^ as positive control. Each graph shows the average dynamic hysteresis loop of a surfactant formulation obtained during the first (black circles), second (red squares), fifth (green triangles) and tenth (inversed yellow triangles) cycle. DP-2L, DP-3L, DP-PA and Curosurf ^®^ needed an area compression of approximately 30% to reach a minimum surface tension of 1 mN/m in the first cycle. Values are means of at least four experiments, SEM values were omitted here for clarity.

First, second, fifth and tenth surface tension-area relations produced by dynamic cycles (20 cycles/min) are shown in Figure 4. The dry powders DP-2L, DP-3L, and DP-PA ( Figure 4) and the clinical surfactant Curosurf ^®^ ( Figure 4) all needed an area compression of approximately 30% to reach a minimum surface tension of 1 mN/m in the first cycle. Dynamic cycling showed a wide difference between the compression and expansion curves for both DP-2L and Curosurf ^®^, with a relatively smaller hysteresis area for DP-PA and an even smaller hysteresis area for DP-3L. The lipid control (DP-C) ( Figure 4) failed to reach low surface tension values as seen during quasi-static cycling in Figure 3.

### *In vivo* experiments: surfactant-deficient rabbits

**Figure 5.**
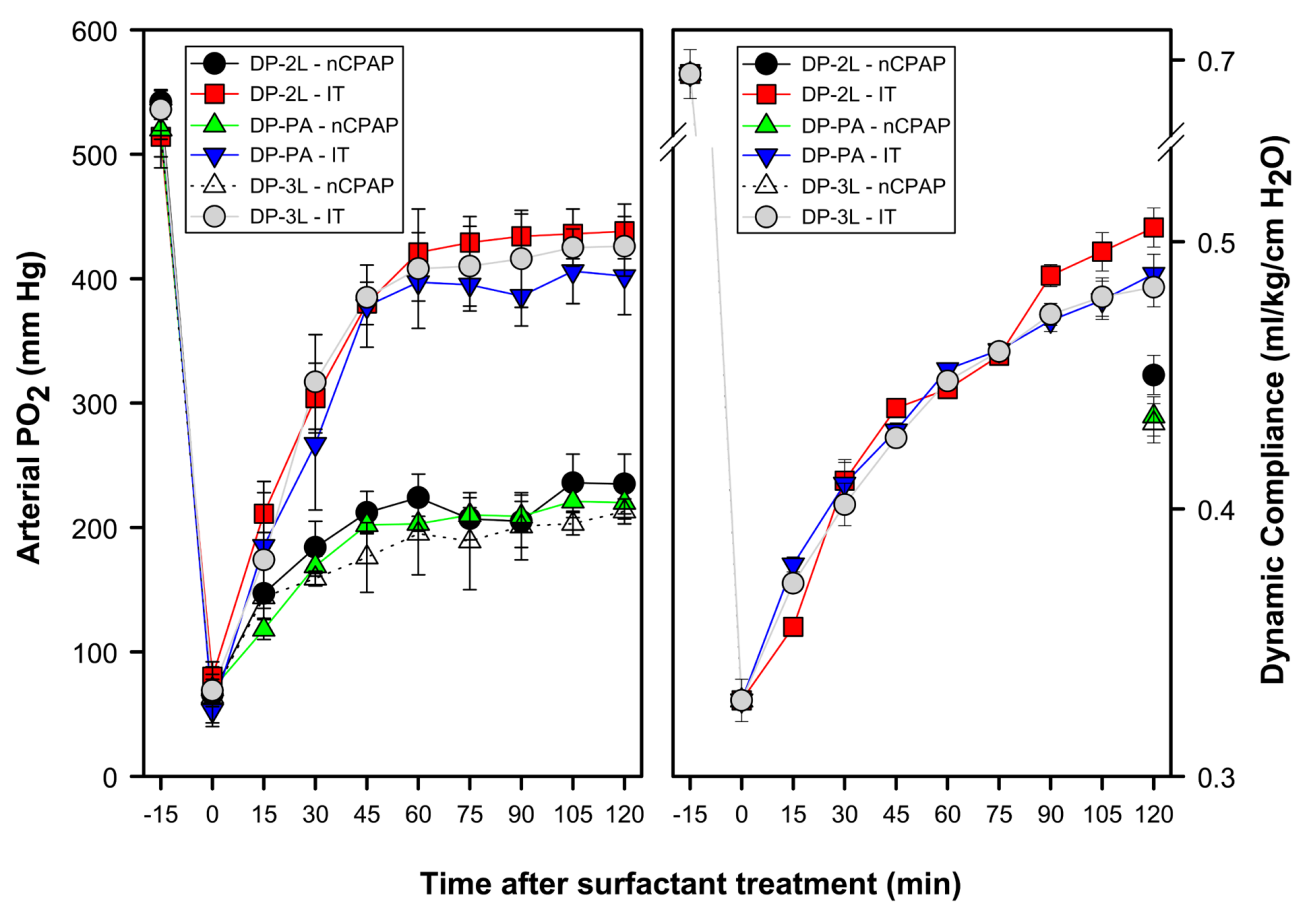
Arterial oxygenation and lung function in lavaged, surfactant-deficient young adult rabbits treated with active dry powder (DP) synthetic surfactant by intratracheal (IT) or nasal continuous positive airway pressure (nCPAP) aerosol delivery. Oxygenation (arterial PO _2_ in mmHg) and lung compliance (mL/kg/cm H _2_O) values of DP-2L, DP-PA and DP-3L surfactants administered IT were compared with these measures obtained after nCPAP delivery. Surfactant (100 mg of lipids/kg) was administered at time 0. Values are mean ± SEM. Oxygenation in the IT groups increased from 10–16% to 75–85% and lung compliance from 46–52% to 69–71% of baseline values measured prior to lung lavage. In the nCPAP groups oxygenation increased from 12–13% to 41–43% and lung compliance from 44–50% to 63–67% of baseline values measure prior to lung lavage. Differences in oxygenation and lung compliance between DP-2L, DP-PA and DP-3L surfactant given IT were not statistically significant and the same held true for DP-2L, DP-PA and DP-3L surfactant given via nCPAP. Differences in oxygenation and lung compliance between IT and nCPAP aerosol delivery were statistically significant for each of the three DP synthetic surfactants (p<0.05).

A total of 30 surfactant-deficient rabbits (mean weight 1.3 ± 0.02 kg) received 100 mg lipids/kg bodyweight of one of the three DP synthetic lung surfactant containing SMB via intratracheal (IT) or nCPAP aerosol delivery. Of the 30, 13 rabbits underwent IT aerosol delivery while supported with mechanical ventilation: 6 received DP-2L, 5 DP-PA and 2 DP-3L. IT delivery was followed by continuation of mechanical ventilation and we observed a quick and large improvement in oxygenation and lung compliance after aerosolization of the three DP synthetic surfactants containing SMB (DP-2L, DP-A and DP-3L) ( Figure 5). Oxygenation in the IT groups increased from 10–16% to 75–85% and lung compliance from 46–52% to 69–71% of baseline values measured prior to lung lavage. The surfactant response was comparable to our recent experience with liquid synthetic surfactant preparations containing 3 wt% of SMB or its sulfur-free derivative B-YL or the clinical surfactant Curosurf ^®^^4, 5, 16^. The remaining 17 rabbits received DP synthetic surfactant by aerosol delivery via the nCPAP system: 8 received DP-2L, 6 DP-PA and 3 DP-3L. Nasal CPAP led to a consistent, but smaller increase in oxygenation and dynamic compliance ( Figure 5). Oxygenation in the nCPAP groups increased from 12–13% to 41–43% and lung compliance from 44–50% to 63–67% of baseline values measured prior to lung lavage. Improvement in lung function in the rabbits receiving mechanical ventilation was independent of the active DP synthetic surfactant administered and the differences among the rabbits treated with surfactant while on nCPAP support were not statistically different either. The differences in oxygenation and lung compliance between IT and nCPAP aerosol delivery of DP-2L, DP-PA and DP-3L were statistically significant for each surfactant (p<0.05).

### *In vivo* experiments: preterm lambs

**Figure 6.**
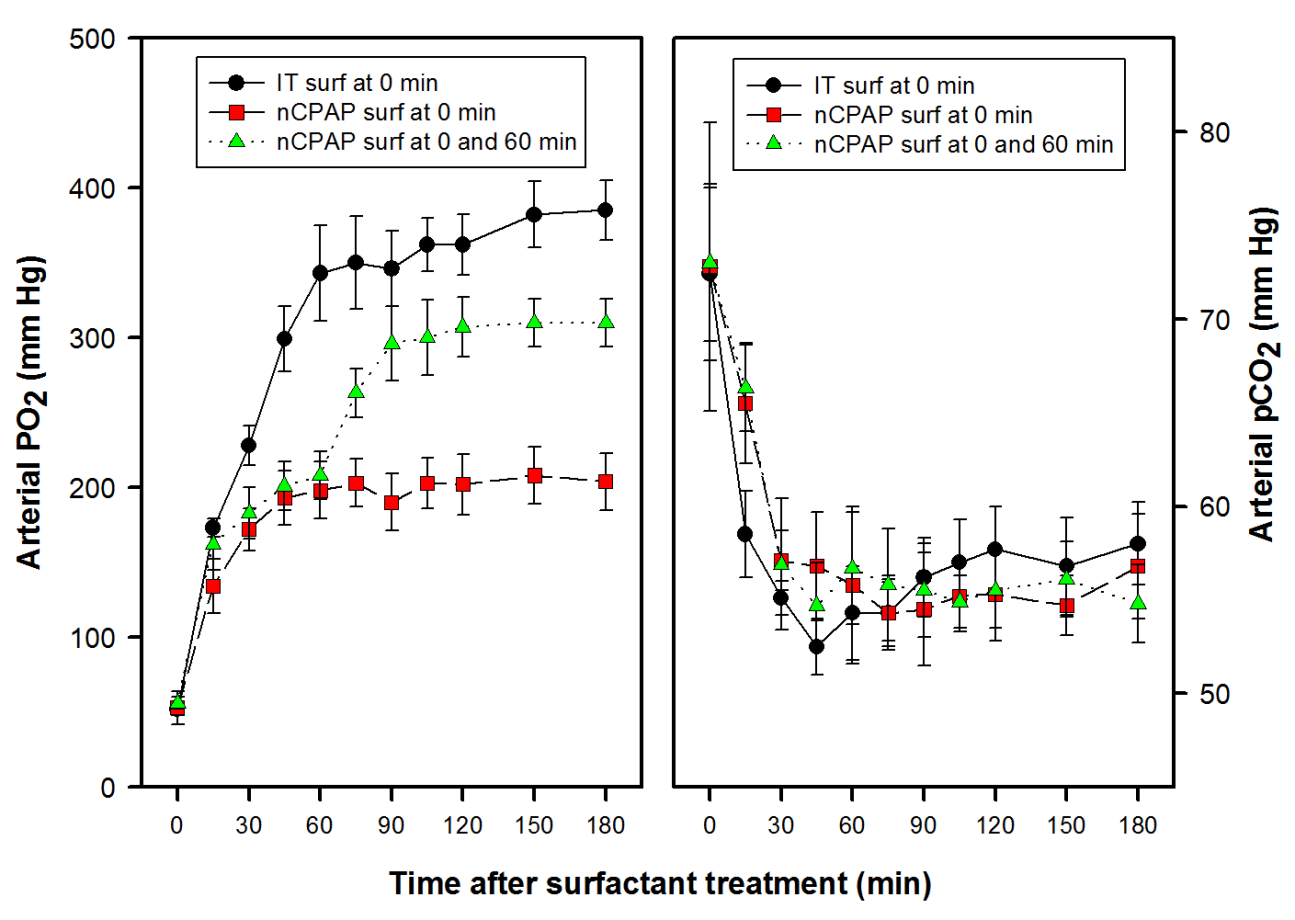
PaO _2_ and PaCO _2_ values in preterm lambs treated with intratracheal (IT) or nasal continuous positive airway pressure (nCPAP) aerosol delivery of synthetic surfactant. A total of 6 preterm lambs were treated with one dose of aerosolized DP-2L surfactant via IT, whereas 7 lambs received 1 dose and six lambs 2 doses of aerosolized DP-2L surfactant via nCPAP. Oxygenation quickly improved after surfactant treatment and PaO _2_ values after IT aerosol delivery exceeded 2 dose nCPAP treatment that, in turn, exceeded 1 dose nCPAP treatment (p<0.02). Mean PaCO _2_ values decreased more slowly and were 55–58 mm Hg (NS) at 3 h after surfactant treatment and this reduction was accompanied by an equivocal increase of pH values (data not shown).

**Figure 7.**
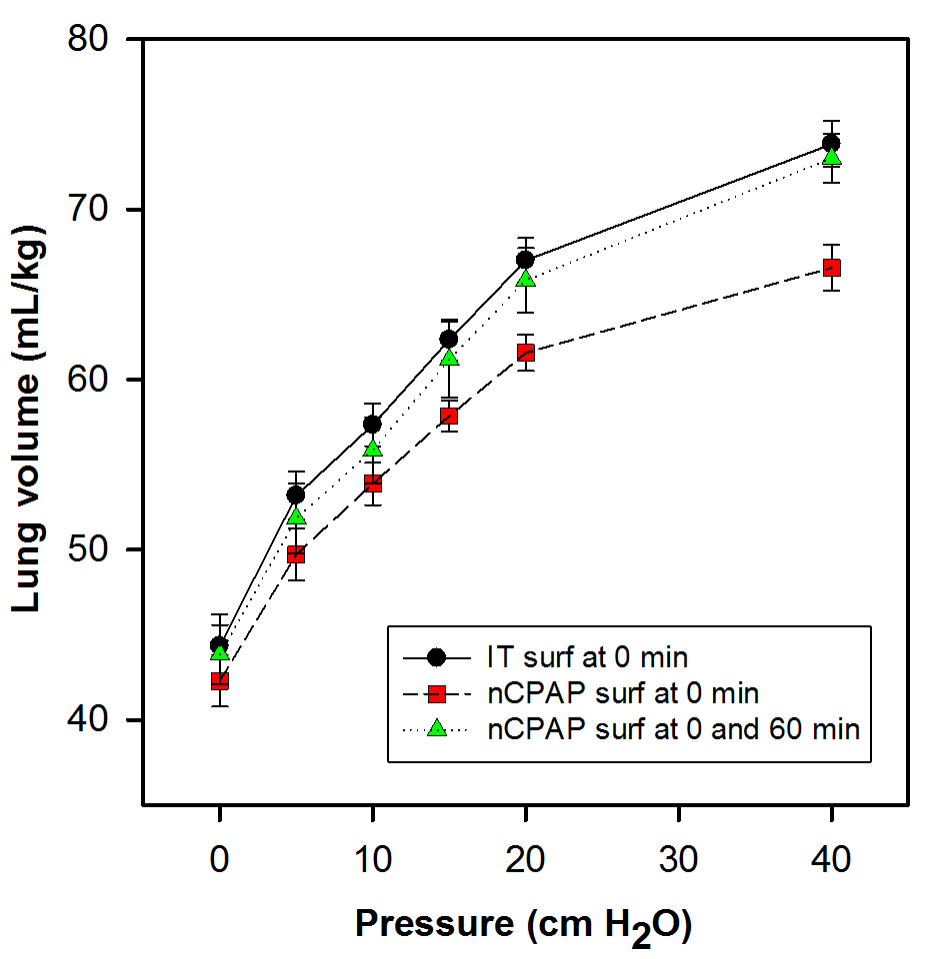
Postmortem pressure-volume curves in preterm lambs treated with intratracheal (IT) or nasal continuous positive airway pressure (nCPAP) aerosol delivery of synthetic surfactant. In total, 6 preterm lambs were treated IT, whereas 7 lambs received 1 dose and six lambs 2 doses of aerosolized DP-2L surfactant via nCPAP. The differences in lung volumes at pressures of 40 and 20 cm H _2_O between IT or two doses of nCPAP surfactant treatment and one dose of nCPAP surfactant were statistically significant (p=0.003 and 0.035, respectively).

A total of 12 pregnant ewes delivered 22 preterm lambs; one ewe carried a single fetus and one ewe had a stillbirth. Of the lambs, three died quickly after birth despite mechanical ventilation and resuscitation efforts, so 19 preterm lambs (weight 3.1 ± 0.2 kg, gestational age 135.5 ± 0.3 days, 11 males and 8 females) received DP synthetic surfactant. In total, 6 lambs were dosed IT and then weaned to nCPAP, 7 lambs received one dose of surfactant while supported with nCPAP and 6 lambs received two doses of surfactant 1 h apart while supported with nCPAP. Based on the available *in vitro* data and experience with surfactant-deficient rabbits, all preterm lambs received DP-2L surfactant at 100 mg lipids/kg bodyweight/dose. All lambs were first weaned to NIPPV before initiating nCPAP support, and, if necessary, were returned for short periods to NIPPV in case of insufficient breathing effort or rising PaCO _2_ values. The response to surfactant treatment was monitored with pulse oximetry and arterial pH and blood gases every 15 min during the 1 ^st^ and 2 ^nd^ hour and every 30 min during the 3 ^rd^ hour after initial surfactant treatment. Oxygenation quickly improved, reaching PaO _2_ values of 385 ± 20 mmHg in 100% oxygen at 3 h after IT aerosol delivery of DP-2L versus 204 ± 19 mm Hg after 1 dose and 310 ± 16 mm Hg after 2 doses of DP-2L administered via the nCPAP system (p<0.02) ( Figure 6). PaCO _2_ values decreased more slowly and were 58.0 ± 2.2, 56.8 ± 2.8 and 54.8 ± 2.1 mm Hg (NS), respectively, at 3 h after surfactant treatment ( Figure 6) and this reduction was accompanied by an equivocal increase of pH values (data not shown). Data from postmortem pressure-volume curves are shown in Figure 7. Only the differences in lung volumes at pressures of 40 and 20 cm H _2_O between IT or two doses of nCPAP surfactant treatment and one dose of nCPAP surfactant were statistically significant (p=0.003 and 0.035, respectively).

## Discussion

Here, we designed and formulated a group of three surface-active dry powder (DP) synthetic lung surfactant preparations with phospholipids DPPC and POPG (7:3 wt:wt) and 3 wt% of the SP-B peptide mimic Super Mini-B (SMB) as surface-active ingredients. These preparations were tested *in vitro* with ATR-FTIR spectrometry and captive bubble surfactometry and *in vivo* in two animal models of surfactant-deficiency, i.e., lavaged young adult rabbits and preterm lambs supported with mechanical ventilation or nCPAP. IT and nCPAP delivery of aerosolized DP synthetic lung surfactant was realized with a proprietary DP delivery device. *In vitro* data showed that the three SMB-containing DP preparations DP-2L, DP-3L, and DP-PA were surface-active. All three surface-active DP synthetic surfactant preparations improved lung function in ventilated and spontaneous breathing surfactant-deficient rabbits receiving nCPAP. The oxygenation response after nCPAP delivery and support amounted to about 50% of the oxygenation response after IT delivery and mechanical ventilation, whereas the improvement in lung compliance was close to 40% in both approaches. Based on the *in vitro* data and rabbit experiments, DP-2L synthetic surfactant was chosen for treatment of the preterm lambs. In the preterm lambs we found a similar difference in the oxygenation response to IT and nCPAP aerosol delivery during non-invasive respiratory support with nCPAP. This difference could be reduced by giving a second dose of DP surfactant during nCPAP support.

Although we used positive and negative controls in the *in vitro* studies, we did not include controls in the current *in vivo* studies as previous work by our group has repeatedly reported the response to lipids alone as negative control and the clinical surfactants Curosurf ^®^ and Infasurf ^®^ as positive controls after IT instillation in lavaged, ventilated young adult rabbits ^4, 5, 9, 16^. After completing the first 6 sheep experiments, we changed the treatment protocol in the preterm lamb studies by adding a group that received two doses of surfactant while supported with nCPAP in order to test whether repeated dosing was a viable and effective approach. The aerosol delivery device we used relies on the use of capsules with a limited amount of DP surfactant, so dosing would take about 10–15 min in the rabbit experiments and 20–30 min in the preterm lamb experiments. Adaptation of this inhaler to facilitate continuous surfactant delivery would enhance the ease of use ^16^.

It is noteworthy that the three SMB-containing DP preparations, administered to animals in the dry state as aerosols, showed good surfactant activities ( Figure 5– Figure 7), which were comparable to earlier *in vivo* findings with intratracheal instillation of liquid surfactant lipids with SMB ^5^. SMB reproduces the topology of the N- and C-terminal domains of SP-B as it contained the α-helical residues 1-25 and 63-78 joined by a custom β-turn –PKGG– and cross-linked with two disulfide bridges ( Table 2) to form an α-helix hairpin. Both homology (I-TASSER) and MD-simulated models of SMB previously demonstrated a globular protein, exhibiting a surface of polar residues and a core of hydrophobic residues that was severely constrained by the disulfide bonds ^8^. Earlier 100-nsec MD simulations also showed that SMB inserted into DPPC/POPG lipid bilayers as a peripheral protein, where the peptide partitions into POPG-enriched domains through selective interactions of its cationic N- and C-helices and the anionic lipid head group ^6^. Notably, a preliminary 499-nsec MD simulation of SMB in surfactant lipid bilayers [i.e., DPPC/POPC/POPG] showed the absence of water within disulfide-linked SMB, and only weakly H-bonded water to several polar residues at the aqueous-bilayer interface ^8^. An additional drying step that temporarily removed only surface-bound water would probably minimally perturb the structure and function of SMB in surfactant lipids, which is supported by FTIR spectroscopy showing α-helix hairpins for SMB in either dry powders ( Figure 1; Table 2) or liquid lipid-peptide solutions ^8^.

Aerosol delivery of surfactant is a new and exciting area of research stimulated by the increasing use of non-invasive respiratory support in preterm infants with RDS ^9, 20^. Early studies with surfactant aerosolization were hampered by the lack of efficient aerosolizers resulting in relatively low lung dosage and limited clinical efficacy. The development of vibrating mesh nebulizers and more insight in their positioning in the ventilation circuit has improved the efficacy of aerosol delivery of surfactant. In a pilot study, Finer *et al.* ^21^ investigated the feasibility and safety of aerosol delivery of Aerosurf ^®^, a synthetic surfactant with the KL4 peptide, with a vibrating mesh nebulizer in preterm infants supported with nCPAP. Ruppert *et al.* ^22^ experimented successfully with DP recombinant surfactant protein-C surfactant (rhSP-C) in three animal models of acute lung injury and Pohlmann *et al.* ^23^ tested a continuous powder aerosolization system with a humidification step for delivery of rhSP-C surfactant delivery via nCPAP in preterm infants. More recently, Milesi *et al*. ^24^ have shown that aerosol delivery of Curosurf, a clinical surfactant, with a customized vibrating mesh nebulizer to preterm lambs receiving nCPAP is safe and improves lung function. A very recent study by Minocchieri *et al*. ^25^ used this approach in preterm infants (gestational ages raging from 29 to 33 weeks) with mild RDS (FiO _2_ between 0.22 and 0.30) and reported similar results as Milesi *et al*. ^24^.

The ultimate goal of this study was to design and develop a low-cost synthetic lung surfactant and a device for aerosol delivery for use in a low technical setting. Using a DP synthetic lung surfactant with an advanced SP-B peptide mimic and a relatively simple low flow inhaler we were able to obtain a pulmonary response in our animal studies that supports translation of this approach to the sketched clinical setting.

## Conclusion

DP synthetic lung surfactant with DPPC and POPG (7:3 wt:wt) and 3 wt% of the SP-B mimic peptide SMB is stable, highly surface active and effective in improving lung function in surfactant-deficient rabbits and preterm lambs.

## Data availability

Raw data are available on OSF: http://doi.org/10.17605/OSF.IO/6295P ^26^

Data are available under the terms of the Creative Commons Zero "No rights reserved" data waiver (CC0 1.0 Public domain dedication).

## References

[1] Polin RA Carlo WA, Committee on Fetus and Newborn, *et al.*: Surfactant replacement therapy for preterm and term neonates with respiratory distress. *Pediatrics.* 2014;133(1):156–163. 10.1542/peds.2013-3443 24379227

[2] Walther FJ Waring AJ Sherman MA: Hydrophobic surfactant proteins and their analogues. *Neonatology.* 2007;91(4):303–310. 10.1159/000101346 17575474

[3] Subramaniam P Ho JJ Davis PG: Prophylactic nasal continuous positive airway pressure for preventing morbidity and mortality in very preterm infants. *Cochrane Database Syst Rev.* 2016; (6):CD001243. 10.1002/14651858.CD001243.pub3 27315509

[4] Walther FJ Gordon LM Waring AJ: Design of Surfactant Protein B Peptide Mimics Based on the Saposin Fold for Synthetic Lung Surfactants. *Biomed Hub.* 2016;1(3): pii: 451076. 10.1159/000451076 28503550 PMC5424708

[5] Walther FJ Waring AJ Hernandez-Juviel JM: Critical structural and functional roles for the N-terminal insertion sequence in surfactant protein B analogs. *PLoS One.* 2010;5(1):e8672. 10.1371/journal.pone.0008672 20084172 PMC2805716

[6] Schwan AL Singh SP Davy JA: Synthesis and activity of a novel diether phosphonoglycerol in phospholipase-resistant synthetic lipid:peptide lung surfactants. *Medchemcomm.* 2011;2(12):1167–1173. 10.1039/C1MD00206F 22530092 PMC3331712

[7] Fezoui Y Weaver DL Osterhout JJ: *De novo* design and structural characterization of an alpha-helical hairpin peptide: a model system for the study of protein folding intermediates. *Proc Natl Acad Sci U S A.* 1994;91(9):3675–3679. 10.1073/pnas.91.9.3675 8170968 PMC43644

[8] Waring AJ Gupta M Gordon LM: Stability of an amphipathic helix-hairpin surfactant peptide in liposomes. *Biochim Biophys Acta.* 2016;1858(12):3113–3119. 10.1016/j.bbamem.2016.09.014 27664499 PMC5096961

[9] Walther FJ Hernández-Juviel JM Waring AJ: Aerosol delivery of synthetic lung surfactant. *PeerJ.* 2014;2:e403. 10.7717/peerj.403 24918030 PMC4045332

[10] Lipp MM Kamerkar A Gilani F: Surfactant formulations for inhalation. WO/2017/223502 A1. Reference Source

[11] Yamaguchi S Hong T Waring A: Solid-state NMR investigations of peptide-lipid interaction and orientation of a beta-sheet antimicrobial peptide, protegrin. *Biochemistry.* 2002;41(31):9852–9862. 10.1021/bi0257991 12146951

[12] Kauppinen JK Moffatt DJ Mantsch HH: Fourier self-deconvolution: A method for resolving intrinsically overlapped bands. *Appl Spectr.* 1981;35:271–276. 10.1366/0003702814732634

[13] Byler DM Susi H: Examination of the secondary structure of proteins by deconvolved FTIR spectra. *Biopolymers.* 1986;25(3):469–487. 10.1002/bip.360250307 3697478

[14] Schürch S Bachofen H Goerke J: A captive bubble method reproduces the *in situ* behavior of lung surfactant monolayers. *J Appl Physiol (1985).* 1989;67(6):2389–2396. 10.1152/jappl.1989.67.6.2389 2606846

[15] Schürch S Bachofen H Possmayer F: Surface activity *in situ*, *in vivo*, and in the captive bubble surfactometer. *Comp Biochem Physiol A Mol Integr Physiol.* 2001;129(1):195–207. 10.1016/S1095-6433(01)00316-6 11369544

[16] Walther FJ Gupta M Gordon LM: A sulfur-free peptide mimic of surfactant protein B (B-YL) exhibits high *in vitro* and *in vivo* surface activities [version 2; referees: 2 approved]. *Gates Open Res.* 2018;2:13. 10.12688/gatesopenres.12799.2 30234192 PMC6139377

[17] Dargaville PA Lavizzari A Padoin P: An authentic animal model of the very preterm infant on nasal continuous positive airway pressure. *Intensive Care Med Exp.* 2015;3(1):51. 10.1186/s40635-015-0051-4 26215815 PMC4512986

[18] Rahmel DK Pohlmann G Iwatschenko P: The non-intubated, spontaneously breathing, continuous positive airway pressure (CPAP) ventilated pre-term lamb: a unique animal model. *Reprod Toxicol.* 2012;34(2):204–215. 10.1016/j.reprotox.2012.05.089 22659287

[19] Surewicz WK Mantsch HH: New insight into protein secondary structure from resolution-enhanced infrared spectra. *Biochim Biophys Acta.* 1988;952(2):115–130. 10.1016/0167-4838(88)90107-0 3276352

[20] Pillow JJ Minocchieri S: Innovation in surfactant therapy II: surfactant administration by aerosolization. *Neonatology.* 2012;101(4):337–344. 10.1159/000337354 22940623

[21] Finer NN Merritt TA Bernstein G: An open label, pilot study of Aerosurf ^^®^^ combined with nCPAP to prevent RDS in preterm neonates? *J Aerosol Med Pulm Drug Deliv.* 2010;23(5):303–309. 10.1089/jamp.2009.0758 20455772

[22] Ruppert C Kuchenbuch T Boensch M: Dry powder aerosolization of a recombinant surfactant protein-C-based surfactant for inhalative treatment of the acutely inflamed lung. *Crit Care Med.* 2010;38(7):1584–1591. 10.1097/CCM.0b013e3181dfcb3b 20400897

[23] Pohlmann G Iwatschenko P Koch W: A novel continuous powder aerosolizer (CPA) for inhalative administration of highly concentrated recombinant surfactant protein-C (rSP-C) surfactant to preterm neonates. *J Aerosol Med Pulm Drug Deliv.* 2013;26(6):370–379. 10.1089/jamp.2012.0996 23421901

[24] Milesi I Tingay DG Lavizzari A: Supraglottic Atomization of Surfactant in Spontaneously Breathing Lambs Receiving Continuous Positive Airway Pressure. *Pediatr Crit Care Med.* 2017;18(9):e428–e434. 10.1097/PCC.0000000000001267 28742723

[25] Minocchieri S Berry CA Pillow JJ: Nebulised surfactant to reduce severity of respiratory distress: a blinded, parallel, randomised controlled trial. *Arch Dis Child Fetal Neonatal Ed.* 2018; pii: fetalneonatal-2018-315051. 10.1136/archdischild-2018-315051 30049729 PMC6764249

[26] Walther FJ: Aerosol delivery of dry powder synthetic lung surfactant. *Open Science Framework.* 2018.

