# Supplementary material for: Aerosol delivery of dry powder synthetic lung surfactant to surfactant-deficient rabbits and preterm lambs on non-invasive respiratory support
Source: Gates Open Res. 2019 Mar 14;3:6. Originally published 2019 Jan 14. [Version 2] doi: 10.12688/gatesopenres.12899.2 (PMC6480449; doi:10.12688/gatesopenres.12899.2)
Supplement: Peer review history

# Peer review: Reviewer response for version 2

**DOI:** 10.21956/gatesopenres.14047.r26998

**Published:** 2019-03-20

**Authors:** Rangasamy Ramanathan

**Affiliations:** 1 Keck School of Medicine, Division of Neonatology, Department of Pediatrics, Los Angeles County+USC Medical Center, University of Southern California (USC), Los Angeles, CA, USA

I approve version 2.

I have read this submission. I believe that I have an appropriate level of expertise to confirm that it is of an acceptable scientific standard.

# Peer review: Reviewer response for version 1

**DOI:** 10.21956/gatesopenres.13995.r26916

**Published:** 2019-02-28

**Authors:** Annelise E. Barron, Jennifer Lin

**Affiliations:** 1 Stanford University, Stanford, CA, USA; 2 Department of Bioengineering, Stanford University, Stanford, CA, USA

In this study, the authors show that a dry powder surfactant formulation containing an SP-B analogue, when delivered as an aerosol using non-invasive respiratory support (nasal continuous positive airway pressure or nCPAP), can significantly improve oxygenation and lung function in surfactant-deficient animals particularly when followed up with a second dose. This reviewer would recommend this manuscript for indexing once minor comments below are addressed.

 1) In the Introduction, the authors cite their previous publication (Ref 9) that investigated aerosol delivery of liquid formulations of synthetic lung surfactant containing SP-B and SP-B & SP-C peptides in which the authors found that the formulation with both SP-B & SP-C yielded better results than the SP-B alone formulation. In this manuscript, the focus is on dry powder (DP) surfactant formulations containing the SP-B peptide analogue. Were there practical reasons for not including SP-C as well in the DP formulations? Is this planned for future work?

 2) A row in Table 1 reads “5°CC 12 mth”. Should this read “5°C” instead as in the powder stability was investigated at refrigeration temperature for the equivalent of 12 months?

 3) The accelerated stability testing conducted at 40°C for the dry powders could use further explanation in the Methods section. It seems to only be briefly mentioned in Table 1 but powder stability is cited as one of the advantages of this surfactant delivery strategy.

 4) Have the authors investigated whether the DP formulation after accelerated stability testing was still active (e.g., via captive bubble surfactometry)? Might the SP-B peptide have undergone some degree of thermal degradation even as a dry powder?

 5) In Figure 3, are the error bars for the Curosurf data and the minimum surface tension measurements absent or so small as to be contained within the data point markers?

 6) Please explain the rationale behind the uneven DP group sizes chosen for the rabbit study, in particular the much smaller n for DP-3L. For rabbits that underwent intratracheal aerosol delivery, 6 received DP-2L, 5 DP-PA, and 2 DP-3L. For rabbits that underwent delivery via the nCPAP system, 8 received DP-2L, 6 DP-PA, and 3 DP-3L.

We have read this submission. We believe that we have an appropriate level of expertise to confirm that it is of an acceptable scientific standard.

# Response

**Published:** 2019-03-07

**Authors:** Frans Walther

**Affiliations:** LA BioMed at Harbor-UCLA Medical Center, USA

1. In the Introduction, the authors cite their previous publication (Ref 9) that investigated aerosol delivery of liquid formulations of synthetic lung surfactant containing SP-B and SP-B & SP-C peptides in which the authors found that the formulation with both SP-B & SP-C yielded better results than the SP-B alone formulation. In this manuscript, the focus is on dry powder (DP) surfactant formulations containing the SP-B peptide analogue. Were there practical reasons for not including SP-C as well in the DP formulations? Is this planned for future work?

**Response**: Adding a SP-C analogue would indeed be a logical next step.

2. A row in Table 1 reads “5°CC 12 mth”. Should this read “5°C” instead as in the powder stability was investigated at refrigeration temperature for the equivalent of 12 months?

**Response**: Thank you for noticing, corrected.

3. The accelerated stability testing conducted at 40°C for the dry powders could use further explanation in the Methods section. It seems to only be briefly mentioned in Table 1 but powder stability is cited as one of the advantages of this surfactant delivery strategy.

**Response**: Further stability testing is being planned.

4. Have the authors investigated whether the DP formulation after accelerated stability testing was still active (e.g., via captive bubble surfactometry)? Might the SP-B peptide have undergone some degree of thermal degradation even as a dry powder?

**Response**: We have indeed only tested “fresh” DP formulations with captive bubble surfactometry.

5. In Figure 3, are the error bars for the Curosurf data and the minimum surface tension measurements absent or so small as to be contained within the data point markers?

**Response**: The standard errors were small and contained within the data point markers. This info was added to the legend of figure 3.

6. Please explain the rationale behind the uneven DP group sizes chosen for the rabbit study, in particular the much smaller n for DP-3L. For rabbits that underwent intratracheal aerosol delivery, 6 received DP-2L, 5 DP-PA, and 2 DP-3L. For rabbits that underwent delivery via the nCPAP system, 8 received DP-2L, 6 DP-PA, and 3 DP-3L.

**Response**: We started our rabbit experiments with 2 DP surfactants (DP-2L and DP-PA) on hand and obtained DP-3L at a later stage.

# Peer review: Reviewer response for version 1

**DOI:** 10.21956/gatesopenres.13995.r26917

**Published:** 2019-02-25

**Authors:** Rangasamy Ramanathan, Hemananda Muniraman

**Affiliations:** 1 NAL, Obstetrix Medical Group of Phoenix, affiliate of Mednax, Phoenix, AZ, USA; 2 Keck School of Medicine, Division of Neonatology, Department of Pediatrics, Los Angeles County+USC Medical Center, University of Southern California (USC), Los Angeles, CA, USA

We would like to commend the authors on a well-conducted study, reporting *in vivo *and *in vitro *activity of a novel DP surfactant with a synthetic analogue of surfactant protein B, Super Mini-B (SMB).

 The DP surfactant preparations appear to be stable in anhydrous state and yet posses high surface activity. The investigators have also designed a low flow aerosolization chamber (LFAC) to deliver the dry powder synthetic SMB via nCPAP. 

 Extensive *in vitro *testing of 3 different synthetic surfactant formulations, DP-2L, DP-3L and DP-PA comparing with synthetic dry powder without SMB as a negative control and clinical surfactant, Curosurf as positive control and authors report similar surface tension reducing properties similar to Curosurf. The authors went on to perform *in vivo *testing, initially in surfactant deficient young adult rabbits comparing arterial oxygenation and dynamic compliance for all three dry powder formulations delivered via intra-tracheal route and during nCPAP. They report significant improvement in oxygenation and lung compliance when the dry powder is delivered intra-tracheal route as compared to nCPAP, while reporting no significant difference between the 3 formulations given by either route. They then conducted experiments in preterm lambs, delivered at 135 days, comparing changes in PaO _2_and PaCO _2_after DP-2L formulation was administered via intra-tracheal route or during nCPAP (1 or 2 doses) and report significant improvement in oxygenation. Administration of a second dose further improved oxygenation. 

 Overall the manuscript reads well with the methods and results sections presented clearly and succinctly. The results of the study are promising for the development of novel synthetic lung surfactant with a device for aerosol delivery. Even though the intra-tracheal route was shown to have the greatest impact on oxygenation and compliance, 2 doses of SMB administered via nCPAP was reported to improve oxygenation and compliance, further studies are needed to evaluate the potential clinical benefits of SMB and LFAC. 

**Specific comments:**

 Introduction:  

 ‘The realization that chronic lung injury (a.k.a. bronchopulmonary dysplasia, BPD) is the result of chronic inflammation due to tracheal intubation and mechanical ventilation has led to a preference for non-invasive respiratory support with nasal continuous positive airway pressure (nCPAP) in preterm infants.” 

 Suggest adding: 

 The etiology of BPD is multifactorial and although the intubation and mechanical ventilation is associated with higher risk of BPD, extremely preterm infants who have never been intubated or mechanically ventilated can still have BPD (new BPD) ^1^. 

 “This approach would especially be useful in preterm infants born in low-resource settings.”

 Authors correctly discuss the benefits of noninvasive ventilation and surfactant therapy.  Minimizing the duration of invasive ventilation, especially during the transitional period following preterm delivery is key in terms of reducing inflammation and lung injury.  Aerosol delivery of surfactant is truly a non-invasive surfactant treatment (NIST) technique. I think, if surfactant in sufficient amounts could be delivered into the lung via aerosol, this combination of noninvasive ventilation and NIST should improve outcomes for all preterm infants and not just for infants born in resource limited settings. I would consider revising this sentence to include all preterm neonates with RDS.

 Methods: 

 Aerosol delivery: we are not sure as to why the authors called 10 l/min as low flow Aerosolization chamber. Please explain.  May just call it as Aerosolization chamber. 

*In vivo experiments: surfactant-deficient rabbits*

 Please describe the details for providing nCPAP in these rabbits. 

*In vivo experiments: preterm lambs*

 In this section, authors describe surfactant delivery during nCPAP. However, the nasal prongs were inserted 6-7 cm for CPAP delivery. This is more of a naso-pharyngeal CPAP 9NP-CPAP) rather than nCPAP. In preterm neonates or term neonates, nasal prongs are typically inserted into the nostrils only 0.5 to 1 cm. Please clarify. 

 Discussion:

 “All three surface-active DP synthetic surfactant preparations improved lung function in ventilated and spontaneous breathing surfactant-deficient rabbits.”

 Change to: 

 “All three surface-active DP synthetic surfactant preparations improved lung function in ventilated and spontaneous breathing surfactant-deficient rabbits receiving nCPAP.”

 “A very recent study by Minocchieri *et al*. ^25^ used this approach in preterm infants and reported similar results as Milesi *et al*.”

 This study was done in more mature preterm infants (29 to 33 weeks gestational age) with mild RDS (FiO _2_0.22 to 030)

 Suggest changing to: 

 “A very recent study by Minocchieri *et al*. ^25^ used this approach in preterm infants  (GA 29 to 33 weeks) with mild RDS (FiO _2_between 0.22 to 0.30) and reported similar results as Milesi *et al*.”

 We would have anticipated lower amount of surfactant delivery in smaller animals, like, rabbits (~weight 1 kg) compared to lambs (~3 kg). Authors may want to discuss about the potential for redosing to improve pulmonary function in smaller animals. 

 Although the investigators mention in their discussion, not including clinical surfactant as a control, following results of their previous study in young adult rabbits, comparison of DP-2L administered via nCPAP with Curosurf administered via intra-tracheal route would have provided important information for future clinical application of SMB delivered by aerosol.

We have read this submission. We believe that we have an appropriate level of expertise to confirm that it is of an acceptable scientific standard.

## References

[1] JobeAHBancalariE: Bronchopulmonary dysplasia. *Am J Respir Crit Care Med*.2001;163(7) : 10.1164/ajrccm.163.7.2011060 1723-9 10.1164/ajrccm.163.7.2011060 11401896

# Response

**Published:** 2019-03-07

**Authors:** Frans Walther

**Affiliations:** LA BioMed at Harbor-UCLA Medical Center, USA

1. Introduction: ‘The realization that chronic lung injury (a.k.a. bronchopulmonary dysplasia, BPD) is the result of chronic inflammation due to tracheal intubation and mechanical ventilation has led to a preference for non-invasive respiratory support with nasal continuous positive airway pressure (nCPAP) in preterm infants.” Suggest adding: The etiology of BPD is multifactorial and although the intubation and mechanical ventilation is associated with higher risk of BPD, extremely preterm infants who have never been intubated or mechanically ventilated can still have BPD (new BPD). 

**Response**: We agree with the reviewer and rephrased this sentence.

2. “This approach would especially be useful in preterm infants born in low-resource settings.”

Authors correctly discuss the benefits of noninvasive ventilation and surfactant therapy.  Minimizing the duration of invasive ventilation, especially during the transitional period following preterm delivery is key in terms of reducing inflammation and lung injury.  Aerosol delivery of surfactant is truly a non-invasive surfactant treatment (NIST) technique. I think, if surfactant in sufficient amounts could be delivered into the lung via aerosol, this combination of noninvasive ventilation and NIST should improve outcomes for all preterm infants and not just for infants born in resource limited settings. I would consider revising this sentence to include all preterm neonates with RDS.

**Response**: Our starting point for this research was to develop a synthetic surfactant treatment for use in low technical settings. We agree with the reviewer and decided to remove this sentence.

3. Aerosol delivery: we are not sure as to why the authors called 10 l/min as low flow Aerosolization chamber. Please explain.  May just call it as Aerosolization chamber. 

**Response**: We wrote “10 L/min or less” because we usually started with a higher flow to get the surfactant capsule spinning and then reduced the flow as far as possible. We changed the text therefore to “(4-10 l/min)”.

4. In vivo experiments: surfactant-deficient rabbits. Please describe the details for providing nCPAP in these rabbits. 

**Response**: We added the following text to this section: “using a F&P bubble CPAP system (Fisher & Paykel Healthcare Inc., Irvine, CA 92618) with short home-made nasal prongs and insufflation of surfactant with the LFAC device at its lowest possible flow setting via the nCPAP system ^9^.”

5. In vivo experiments: preterm lambs. In this section, authors describe surfactant delivery during nCPAP. However, the nasal prongs were inserted 6-7 cm for CPAP delivery. This is more of a naso-pharyngeal CPAP (NP-CPAP) rather than nCPAP. In preterm neonates or term neonates, nasal prongs are typically inserted into the nostrils only 0.5 to 1 cm. Please clarify.

**Response**: We inserted the home-made prongs deeper into the nose of the preterm lambs to get a better fixation and optimize aerosol delivery based on the work by Dargaville et al. (reference 17) and added this reference.   

6. “All three surface-active DP synthetic surfactant preparations improved lung function in ventilated and spontaneous breathing surfactant-deficient rabbits.” Change to: “All three surface-active DP synthetic surfactant preparations improved lung function in ventilated and spontaneous breathing surfactant-deficient rabbits receiving nCPAP.”

**Response**: Text changed as suggested.

7. “A very recent study by Minocchieri *et al*. ^25^ used this approach in preterm infants and reported similar results as Milesi *et al*.” This study was done in more mature preterm infants (29 to 33 weeks gestational age) with mild RDS (FiO _2_0.22 to 030). Suggest changing to: “A very recent study by Minocchieri *et al*. ^25^ used this approach in preterm infants (GA 29 to 33 weeks) with mild RDS (FiO _2_between 0.22 to 0.30) and reported similar results as Milesi *et al*.”

**Response**: Text changed as suggested.

8. We would have anticipated lower amount of surfactant delivery in smaller animals, like, rabbits (~weight 1 kg) compared to lambs (~3 kg). Authors may want to discuss about the potential for redosing to improve pulmonary function in smaller animals. 

**Response**: Redosing might indeed have improved the surfactant response in the lavaged, surfactant-deficient rabbits, but redosing was not tested in the rabbit model.

9. Although the investigators mention in their discussion, not including clinical surfactant as a control, following results of their previous study in young adult rabbits, comparison of DP-2L administered via nCPAP with Curosurf administered via intra-tracheal route would have provided important information for future clinical application of SMB delivered by aerosol.

**Response**: Please consult reference 16 for the surfactant response on intratracheal Curosurf administration in lavaged, surfactant-deficient rabbits.

# Peer review: Reviewer response for version 1

**DOI:** 10.21956/gatesopenres.13995.r26898

**Published:** 2019-02-13

**Authors:** Noah H. Hillman

**Affiliations:** 1 Division of Neonatology, Cardinal Glennon Children’s Hospital, Saint Louis University, St. Louis, MO, USA

Walther *et al.* present very interesting *in vivo* and *in* *vitro* data on three dry powder combinations of phospholipids with the synthetic surfactant protein Super Mini-B (SMB). The manuscript builds upon the groups previous publications of liquid forms of the SMB. By creating a dry version that is clinically stable at room temperature and can be easily aerosolized, they can provide the beneficial effects of surfactant in limited resource environments.

 The authors demonstrate that the three surfactant/phospholipid mixtures were stable at high temperatures for multiple months, with the DP-2L combination the most stable. They then demonstrated that the DP-2L mixture has the closest dynamic cycling curves to Curosurf. 

 The authors used standard protocols for saline lavaged rabbits and preterm lambs that were appropriate for the studies they conducted. The rabbits demonstrated improvement in oxygenation and compliance with all three mixtures, with less improvement in rabbits on CPAP (likely due to less of the surfactant mixture getting into the lungs). Based on the stability data and the responses in the rabbits, the DP-2L mixture was chosen to be tested preterm sheep. The addition of a second dose of aerosolized surfactant at 1 hour provided additional benefit to the lambs.

 The lack of the Curosurf or phospholipid only groups in the animal studies makes placing the response to the aerosolized mixtures more difficult. The authors addressed this concern in the discussion. Evaluation of their previous publication ^1^ demonstrates no response to phospholipid alone over time of ventilation, and a similar response to the IT instillation of the SMB compound and Curosurf. Based on this data, the conclusions they draw within the manuscript are correct and the aerosolized dry surfactant is nearly as effective a commercially available surfactant given via an endotracheal tube.

**Minor suggestions:**

 Abstract: The methods section of the abstract about the animal studies is a little confusing. Reading the methods in the manuscript, it is clearer that some of the animals were intubated and the dry powder aerosolized to assure it made it to the lungs of the animals. The word “active” in the conclusion sentence of abstract could be removed.

 Introduction: Since BPD can occur in infants without mechanical ventilation and is likely a combination of multiple factors, the phrase “is the result of” in the introductory paragraph could be altered.

 Table 1: There are two C after 5 degrees.

 Figure 2: The figure is confusing as the diagrams for the 3 spectral plots look like they have difference degrees of absorption with A absorbing more than B and more than C. The graphs could be superimposed on one another with a different line type for each mixture. Then a legend could be put on the graph that demonstrates the name of the mixture. The information could also be placed in Table 2 alongside the secondary data structure.

 Figure 3: Curosurf data does not have any error bars, whereas other types of treatment have error bars. Were the values identical on repeat measures?

 Figure 4: For ease of reader, the authors could consider changing DP-C to Control or Negative Control in the figure.

 Figure 5/6: Statistics between the group would be useful on the figures to assist readers. They are available in the figure legend and the manuscript text.

 Figure 5: The straight lines for the CPAP animals could be removed and the markers left at the 120 minute position since it is unlikely the compliance improved in a linear fashion between the two points of assessment of compliance in these spontaneously breathing animals.

I have read this submission. I believe that I have an appropriate level of expertise to confirm that it is of an acceptable scientific standard.

## References

[1] WaltherFJGuptaMGordonLMWaringAJ: A sulfur-free peptide mimic of surfactant protein B (B-YL) exhibits high in vitro and in vivo surface activities. *Gates Open Res*.2018;2: 10.12688/gatesopenres.12799.2 13 10.12688/gatesopenres.12799.2 30234192PMC6139377

# Response

**Published:** 2019-03-07

**Authors:** Frans Walther

**Affiliations:** LA BioMed at Harbor-UCLA Medical Center, USA

1. Abstract: The methods section of the abstract about the animal studies is a little confusing. Reading the methods in the manuscript, it is clearer that some of the animals were intubated and the dry powder aerosolized to assure it made it to the lungs of the animals. The word “active” in the conclusion sentence of abstract could be removed.

**Response**: We removed the word “active” from the abstract.

2. Introduction: Since BPD can occur in infants without mechanical ventilation and is likely a combination of multiple factors, the phrase “is the result of” in the introductory paragraph could be altered.

**Response**: We agree with the reviewer and rephrased this sentence.

3. Table 1: There are two C after 5 degrees.

**Response**: Thank you for noticing, corrected.

4. Figure 2: The figure is confusing as the diagrams for the 3 spectral plots look like they have difference degrees of absorption with A absorbing more than B and more than C. The graphs could be superimposed on one another with a different line type for each mixture. Then a legend could be put on the graph that demonstrates the name of the mixture. The information could also be placed in Table 2 alongside the secondary data structure.

**Response**: We redid figure 2, thereby superimposing the graphs on one another and changing the legend accordingly.

5. Figure 3: Curosurf data does not have any error bars, whereas other types of treatment have error bars. Were the values identical on repeat measures?

**Response**: The standard errors were small and contained within the data point markers. This info was added to the legend of figure 3.

6. Figure 4: For ease of reader, the authors could consider changing DP-C to Control or Negative Control in the figure.

**Response**: We considered it, but this might lead to confusion when readers consult reference 16 for the data on intratracheal administered Curosurf and lipid controls.

7. Figure 5/6: Statistics between the group would be useful on the figures to assist readers. They are available in the figure legend and the manuscript text.

**Response**: To avoid overcrowding, we described the data and report statistics in the legends of figures 5 and 6.

8. Figure 5: The straight lines for the CPAP animals could be removed and the markers left at the 120 minute position since it is unlikely the compliance improved in a linear fashion between the two points of assessment of compliance in these spontaneously breathing animals.

**Response**: Figure 5 was changed accordingly.
